# Comparison of the effects of neuromuscular electrical stimulation plus conventional treatment to those of conventional treatment alone in patients with fibromyalgia: A randomized controlled study

**DOI:** 10.1097/MD.0000000000043933

**Published:** 2025-08-15

**Authors:** Muhammet Sahin Elbasti, Songul Baglan Yentur

**Affiliations:** a Department of Physical Medicine and Rehabilitation, Elazig Fethi Sekin City Hospital, Elazig, Turkey; b Department of Physiotherapy and Rehabilitation, Faculty of Health Sciences, Firat University, Elazig, Turkey.

**Keywords:** conventional treatment, fibromyalgia syndrome, neuromuscular electrical stimulation

## Abstract

**Background:**

The aim of this study was to investigate the effects of neuromuscular electrical stimulation (NMES) in addition to the conventional treatment (CT) on pain, quality of life, sleep quality and posture, and compare with CT alone.

**Methods:**

This study included 40 female patients with fibromyalgia. Pain intensity, sleep quality, quality of life and posture were evaluated with visual analog scale, Pittsburg Sleep Quality Index, Fibromyalgia Impact Questionnaire and New York Posture Rating Chart, respectively. Patients were divided in 2 groups randomly. First group was applied NMES + CT and second group was applied CT alone. The interventions were lasted for 6 weeks.

**Results:**

No statistically significant differences was found between groups in demographic variables. After treatment, a statistical difference was found between the 2 groups only in pain intensity (*P* = .015). Significant differences were concluded in comparison of before and after treatment in both groups (*P* < .05). In addition, significant difference between the groups in pain (*P* = .006) and posture (*P* = .014) in the comparison of delta (Δ) measurements between groups.

**Conclusion:**

Additional NMES application was found superior on pain and posture compared to CT alone in patients with fibromyalgia syndrome. Adding NMES to the routine treatment may increase the success of fibromyalgia syndrome rehabilitation.

## 1. Introduction

Fibromyalgia syndrome (FMS) is a complex chronic pain disorder marked by diffuse musculoskeletal pain, fatigue, sleep irregularities, and cognitive symptoms. The diagnostic framework has evolved with the 2010 revision by the American College of Rheumatology, which emphasizes symptom severity and distribution over the traditional tender point count. The disease most commonly affects women aged 40 to 60 years. Although the pathogenesis is not known exactly, central sensitization is held responsible. The pain observed in FMS typically lasts longer than 3 months. Approximately 75% of patients with FMS have sleep disorders. Patients report that they wake up frequently at night and wake up tired in the morning.^[1]^ Pain and other symptoms associated with FMS interfere with daily functions, work and social activities, leading to a decrease in quality of life. Symptoms of FMS cause physical fitness impairment. A recent study evaluating physical condition by accelerometry was reported lower physical activity in FMS patients compared to healthy individuals.^[2]^ It is assumed that physical deconditioning leads to enhanced muscle ischemia and increased peripheral sensitization. As a result, it may contribute to the development of central sensitization.^[3]^

It is known that patients with FMS have decreased muscle strength compared to healthy controls. A mean reduced muscle strength of 39% has been reported compared to healthy subjects.^[4]^ Possible mechanisms for decreased strength in FMS patients can be listed as follows: structural changes in muscle fibers, altered neuromuscular control mechanisms, impaired blood circulation and regulation of growth and energy metabolism, and low amounts of physical activity. FMS causes structural changes in muscle fibers, abnormalities in microcirculatory capillaries and irregularities in muscle metabolism.^[5]^ It is known that losses in muscle strength may cause microtrauma due to strain during physical activity.^[6]^ Therefore, studies investigating the effects of exercise in FMS have generally emphasized that aerobic and relaxation exercises are effective on function and pain. It was thought that muscle strengthening activities would lead to muscle damage and increase pain.^[7]^ However, later studies showed that muscle strengthening did not cause such damage; on the contrary, it had ameliorative effects on symptoms.^[6,7]^ However, studies on muscle strengthening in FMS patients are limited.

Neuromuscular electrical stimulation (NMES) produces contraction by stimulating nerve fibers providing innervation in healthy muscle and muscle fibers in denervated muscle.^[8]^ NMES applied to normal muscle provides an increase in strength. The action potential coming from the central nervous system is mimicked by NMES to produce contraction in the muscle.^[9]^ It stimulates muscle contractile elements and is aimed to produce tetanic contraction.^[10]^ Also, inhibitory pathways descending from the midbrain and brainstem are activated with electrical stimulation, thus preventing the excitability of nociceptive neurons in the spinal cord, which leads to a reduction in pain. Transcutaneous electrical nerve stimulation (TENS) was mostly practiced for pain. However, studies investigating the effects of TENS on pain are contradictory. A systematic review and meta-analysis examining the effects of TENS in combination with other treatments or alone in patients with FMS was reported reduced pain, but no significant changes in quality of life and fatigue. No significant improvement in pain was found in another study in which TENS was applied alone.^[10]^ Conventional physiotherapy including exercise, TENS, therapeutic ultrasound, laser and magnetotherapy in patients with FMS was reported decreased pain and improved functional status.^[11]^ However, although muscle weakness has been described in patients with FMS, there are no studies on the effects of NMES application in these patients to the authors’ knowledge. The additive effects of NMES might be attributed to its dual mechanisms: enhancing neuromuscular activation and inhibiting spinal nociceptive neurons, thereby complementing the benefits of conventional physiotherapy. We hypothesized that people with fibromyalgia who receive NMES as an adjunct to conventional therapy will have greater reductions in pain intensity than those who receive conventional therapy alone. This study aimed to investigate whether integrating NMES into a conventional physiotherapy program would lead to greater improvements in pain severity, physical function, sleep quality, and postural alignment compared to conventional therapy alone in individuals with FMS.

## 2. Patients and method

The sample size and power required for the study were calculated with the G*Power Ver.3.1.9.4 program. In the power analysis based on the results of the comparison of pain mean scores in a study conducted by Ustabasioglu^[12]^ with the condition of α = 0.05 risk, 1‐α = 0.95 accuracy rate and the effect size 1.26 actual power: 0.95, it was concluded that a minimum of 19 people should participate in each group. Randomization was performed by taking 10% more in terms of possible sample loss, with 21 people for each group and 42 people in total. However, since 2 patients did not want to continue the study and wanted to leave, the study was completed with a total of 40 people. Participants were recruited from the outpatient physical therapy and rehabilitation clinic of Medical Hospital between July and December 2024. Eligible patients were identified by the attending clinicians and invited to participate during their routine visits. The study included 40 female patients diagnosed with FMS who met the inclusion criteria and agreed to participate in the study. Patients diagnosed with FMS were assigned to NMES + conventional treatment (CT) and CT alone by randomization. For randomization, a single group column between 1 and 40 was created in the system using the Random Integer Generator method in the Numbers subheading of the random.org website. Considering the numbers 1 and 2 in the column, NMES + CT group and CT group were randomly assigned to numbers 1 and 2. Which number was in which group was determined by drawing lots at the beginning of the study. Ethics committee was obtained before starting the study from the Ethics Committee of Firat University Non-Interventional Ethics Committee (2024/10-01) and written informed consent was obtained from the patients during the study. This study conforms to CONSORT guidelines and reports the required information accordingly (see Supplementary Checklist, Supplemental Digital Content, https://links.lww.com/MD/P688). The study was designed in accordance with the Declaration of Helsinki.

The study included female patients aged 18 to 65 years, diagnosed with FMS, who agreed to participate in the study and whose medical treatment was stable within the last 3 months. Patients with a diagnosis of pregnancy, malignancy or infection, severe spinal stenosis, concomitant systemic inflammatory rheumatic disease, history of surgery within the last 3 months and regular exercise habits were excluded. In addition, patients who voluntarily discontinued participation during the treatment program, patients who did not attend 50% or more of the treatment program, patients who did not attend 3 consecutive sessions of the treatment program, and patients who left our province due to change of address were excluded from the study.

Firstly, socio-demographic data (age, height, weight, marital status, employment status, and smoking) were questioned and recorded. Then pain intensity, sleep quality, quality of life, and posture were evaluated in all patients. The evaluation methods were repeated before and after treatment. After the evaluation, all patients were taught neck range of motion exercises, upper trapezius stretching exercise and posture exercises. In the NMES + CT group, NMES was applied to the upper trapezius muscle for 20 minutes 5 days a week for 6 weeks in addition to conventional treatment.

## 3. Interventions

### 3.1. Conventional treatment group

Physiotherapy and rehabilitation methods applied in FMS treatment were applied to the conventional treatment group. Electrotherapy methods, infrared application and stretching, range of motion, strengthening and posture exercises were applied. The treatment lasted approximately 45 minutes. The treatment was applied 5 days a week for 4 weeks. Among the electrotherapy methods, conventional TENS (current frequency 100 Hz and current passage time 100 µs) and therapeutic ultrasound (1.5 W/cm^2^, 1 mHz head for 5 minutes) were applied. Infrared application was applied 20 cm away from the cervical region.

### 3.2. NMES + conventional treatment group

In the NMES + CT group, a conventional treatment program was applied for 45 minutes as in the conventional treatment group. In addition, NMES was applied to the upper trapezius muscle for 20 minutes.

NMES applications were performed with a Compex 3 (Compex Medical SA, Ecublens, Switzerland) model, portable and programmable electrostimulator unit in rehabilitation mode and under the supervision of an expert physiotherapist experienced in the device and application. The NMES program was applied with a 4-channel device with a functional stimulation capacity of 120 mA, pulse duration 400 μs and pulse frequency 150 Hz. In the program, self-adhesive plastic electrodes with a thickness of 2 mm were used on the skin surface. Electrodes with a 25 cm^2^ (5 cm × 5 cm) measuring surface were placed on a motor area in the center of the trapezius muscle in the back. The current intensity was adjusted at a dose that would cause muscle contraction and that the patient could tolerate, and NMES was applied for 20 minutes with the participants lying face down on the stretcher.

## 4. Outcome measurements

### 4.1. Pain assessment

Pain intensity was evaluated according to visual analog scale. Patients will be asked to mark the intensity of pain on a 10 cm line. Zero means no pain and 10 means unbearable pain.^[13]^

### 4.2. Quality of life assessment

The Fibromyalgia Impact Questionnaire was used to evaluate quality of life which consists of 10 items. The first item includes daily activity questions scored on a Likert scale from 0 to 3 (always able to do–never able to do). The 10 items are added together to calculate a total score.^[14]^

### 4.3. Sleep quality assessment

Sleep quality was assessed with the Pittsburg Sleep Quality Index (PSQI). It consists of 24 questions in total. Nineteen questions are self-report questions and 5 questions are answered by a spouse or roommate. The last 5 questions are not taken into account in the scoring of the PSQI and are used only for clinical information. The PSQI produces 7 “component” scores: subjective sleep quality, sleep latency, duration, habitual sleep efficiency, sleep disturbances, use of sleep medication and daytime dysfunction. The sum of the 7 component scores constitutes the total PSQI score. A total PSQI score between 0 and 4 points is considered “good sleep quality,” while a score between 5 and 21 points is considered “poor sleep quality.” The diagnostic sensitivity and specificity of the PSQI were found to be 89.6% and 86.5%, respectively. The validity and reliability study in Turkey was conducted by Ağargün, Anlar, and Kara.^[15]^

### 4.4. Posture assessment

Posture was assessed with the New York Posture Rating Chart. This test evaluates the body in 13 different parts in terms of posture changes. A score of 5 is given if the person’s posture is correct, 3 if it is moderately impaired and 1 if it is severely impaired. The total score obtained as a result of the test varies between 13 and 65. A high score means that the posture is good.^[16]^

### 4.5. Statistical analysis

All statistical evaluations were performed with Statistical Packages for Social Sciences (SPSS) Version 22.0 for MS Windows program. Shapiro–Wilk test was applied to determine whether the data were normally distributed. The obtained data were analyzed using parametric or nonparametric statistical methods. Descriptive values were expressed as number, percentage, minimum, maximum, mean ± standard deviation. Pearson chi-square test was used for categorical variables, independent *t* test and Mann–Whitney *U* test were used for continuous variables. Wilcoxon test, which is a nonparametric dependent group comparison statistic, and paired sample test statistics were used for parametric comparisons. To analyze in which group the improvement was more pronounced, the difference between the parameters examined between the 2 groups was examined. *P* < .05 was considered statistically significant for the parameters analyzed between the 2 groups.

## 5. Results

Demographic characteristics of the patients were summarized in Table 1. There was no statistically significant difference between the demographic variables of the groups (*P* > .05).

**Table 1:** Demographic variables of treatment groups.

Parameters		NMES + CT group (n = 20)	CT group (n = 20)	*P*
Age/yr		40.20 ± 8.79 (25.00–58.00)	39.70 ± 8.94 (24.00–54.00)	.859
Height/cm		162.75 ± 4.50 (154.00–170.00)	162.30 ± 4.68 (155.00–172.00)	.758
Weight/kg		71.25 ± 9.22 (53.00–85.00)	70.30 ± 10.86 (53.00–94.00)	.767
BMI/kg/cm^2^		26.93 ± 3.66 (20.32–34.55)	26.65 ± 3.76 (20.45–34.93)	.817
Smoking	No	6 (30%)	2 (10%)	.114
	Yes	14 (70%)	18 (90%)	
Marital status	Married	16 (80%)	17 (85%)	.677
	Single	4 (20%)	3 (15%)	
Education level	Primary school	13 (65%)	10 (50%)	.554
	High school	4 (20%)	4 (20%)	
	University	3 (15%)	6 (30%)	
Occupation	No	17 (85%)	12 (60%)	.077
	Yes	3 (15%)	8 (40%)	

BMI = body mass index, CT = conventional therapy, NMES = neuromuscular electrical stimulation.

The comparison between the 2 groups before and after treatment is summarized in Table 2. There was no difference between the 2 groups in the parameters examined before treatment (*P* > .05). After treatment, a statistical difference was observed between the 2 groups only in pain intensity (*P* = .015) (Table 2). The comparison within the group differences before and after treatment is summarized in Table 3. Statistically significant differences were observed in pain (*P* < .001), quality of life (*P* < .001, sleep quality (*P* < .001) and posture (*P* < .001) in NMES + CT group and pain (*P* < .001), quality of life (*P* < .001), sleep quality (*P* = .001), and posture (*P* < .001) in CT group in comparison to before and after treatment. There was a statistically significant difference between the groups in pain (*P* = .006) and posture (*P* = .014) in the comparison of delta (Δ) measurements between groups (*P* < .05) (Table 4).

**Table 2:** Between-group comparisons before and after treatment.

Before treatment				After treatment		
Parameters	NMES + CT group (n = 20)	CT group (n = 20)	*P*	NMES + CT group (n = 20)	CT group (n = 20)	*P*
FIQ	77.53 ± 8.38 (65.15–94.03)	77.75 ± 5.48 (71.20–88.20)	.640	63.31 ± 6.71 (52.87–79.74)	63.98 ± 7.57 (52.87–83.74)	.547
VAS	7.35 ± 1.18 (6.00–10.00)	7.55 ± 1.31 (5.00–10.00)	.565	5.30 ± 1.30 (4.00–8.00)	6.30 ± 1.17 (4.00–8.00)	**.015**
NYPR	27.10 ± 4.29 (21.0–38.00)	26.70 ± 2.93 (20.00–31.00)	.733	29.45 ± 4.24 (23.00–41.00)	28.15 ± 2.81 (22.00–32.00)	.261
PSQI	9.00 ± 2.95 (3.00–13.00)	8.00 ± 3.07 (3.00–13.00)	.301	7.50 ± 2.41 (3.00–11.00)	7.00 ± 2.29 (3.00–11.00)	.506

Bold values indicate statistically significant results (*P* < .05). CT = conventional therapy, FIQ = Fibromyalgia Impact Questionnaire, NMES = neuromuscular electrical stimulation, NYPR = New York Posture Rating Chart, PSQI = Pittsburgh Sleep Quality Index, VAS = visual pain scale.

**Table 3:** Within-group differences before and after the treatment.

Parameters	NMES + CT group			CT group		
	Before treatment	After treatment	*P*	Before treatment	After treatment	*P*
FIQ	77.53 ± 8.38 (65.15–94.03)	63.31 ± 6.71 (52.87–79.74)	**<.001**	77.75 ± 5.48 (71.20–88.20)	63.98 ± 7.57 (52.87–83.74)	**<.001**
VAS	7.35 ± 1.18 (6.00–10.00)	5.30 ± 1.30 (4.00–8.00)	**<.001**	7.55 ± 1.31 (5.00–10.00)	6.30 ± 1.17 (4.00–8.00)	**<.001**
NYPR	27.10 ± 4.29 (21.00–38.00)	29.45 ± 4.24 (23.00–41.00)	**<.001**	26.70 ± 2.93 (20.00–31.00)	28.15 ± 2.81 (22.00–32.00)	**<.001**
PSQI	9.00 ± 2.95 (3.00–13.00)	7.50 ± 2.41 (3.00–11.00)	**<.001**	8.00 ± 3.07 (3.00–13.00)	7.00 ± 2.29 (3.00–11.00)	**.001**

Bold values indicate statistically significant results (*P* < .05). CT = conventional therapy, FIQ = Fibromyalgia Impact Questionnaire, NMES = neuromuscular electrical stimulation, NYPR = New York Posture Rating Chart, PSQI = Pittsburgh Sleep Quality Index, VAS = visual pain scale.

**Table 4:** Comparison of delta measurements of groups.

Parameters	NMES + CT group (n = 20)	CT group (n = 20)	*P*
ΔFIQ	14.22 ± 6.84 (3.12–32.13)	13.77 ± 6.82 (0.00–26.36)	.838
ΔVAS	2.05 ± 0.75 (1.00–4.00)	1.25 ± 0.78 (–1.00 to 2.00)	**.006**
ΔNYPR	–2.35 ± 1.72 (–5.00 to 3.00)	–1.45 ± 1.14 (–4.00 to 0.00)	**.014**
ΔPSQI	1.50 ± 1.05 (0.00–3.00)	1.00 ± 1.16 (0.00–4.00)	.127

Bold values indicate statistically significant results (*P* < .05). CT = conventional therapy, FIQ = Fibromyalgia Impact Questionnaire, NMES = neuromuscular electrical stimulation, NYPR = New York Posture Rating Chart, PSQI = Pittsburgh Sleep Quality Index, VAS = visual pain scale.

## 6. Discussion

This study was designed to evaluate the effects of NMES in addition to conventional treatment in patients with FMS. The study was found that both conventional treatment and NMES as an adjunct to conventional treatment had positive effects on pain intensity, sleep quality, quality of life and posture. In addition, the additional application of NMES provided additional improvements in pain intensity and posture. NMES treatment was concluded to be feasible in terms of clinical symptoms in patients with FMS.

It was observed that NMES treatment applied in addition to conventional treatment methods had positive effects on pain intensity, function, posture and sleep quality in this study. It is known that patients with FMS have impaired quality of life, posture and sleep quality, decreased sleep effect and increased fatigue compared to healthy subjects.^[17]^ Pharmacologic and non-pharmacologic approaches are used in its treatment. The most recommended non-pharmacologic approach is exercise therapy. Since muscle damage is involved in FMS, it was believed that isometric exercises would increase the severity of pain and aerobic exercises and relaxation training were preferred.^[18]^ There was a belief that muscle strengthening training would increase muscle damage and it was shown that exercise-induced pain during progressive resistance exercise could be prevented by gradually switching to heavier loads.^[19]^ However, studies investigating the effects of NMES application used for muscle reeducation in patients with FMS were not found by the authors. NMES has been widely adopted as a rehabilitation/educational method in both research and clinical settings. Depending on the condition of the stimulated muscle, NMES is used to maintain muscle mass and function during periods of prolonged disuse or inactivity, to restore muscle mass and function after periods of prolonged disuse or inactivity, and to improve muscle function in different healthy populations.^[6]^ Our study is important because it is the first study to examine the effects of NMES application on some symptoms in patients with FMS. No patient spontaneously reported adverse side effects or discomfort during or after NMES application. However, side effects and discomfort were not systematically evaluated in this study. There may be a feeling of discomfort during NMES application. An overlooked feature of NMES is its potential to activate pain inhibition mechanisms through the uncomfortable sensations of stimulation. Conditioned pain modulation, known as ‘pain inhibiting pain’, may be effective in this mechanism. However, exercise-induced hyoalgesia and long-term depression may also be effective mechanisms. Inhibition of transmission of painful stimuli may result from long-term depression of dorsal horn synapses.^[20]^ In addition, activation of large diameter Aβ fibers may also be involved in pain inhibition by electrical stimulation. As in the gate control theory, pain inhibition may have occurred as a result of some changes in the substantia gelatinosa. Finally, the brainstem pain modulation system may have played a role in pain inhibition. Endogenous opioids produce inhibition via serotonin and noradrenaline.^[21]^ Although NMES is primarily used for muscle strengthening and causes discomfort during application, it is also effective in pain inhibition through the mechanisms mentioned above. However, pain induced during NMES may further improve function.^[22]^ In our study, the fact that additional NMES was effective on pain in women with FMS may have been provided by these mechanisms. Studies have shown a relationship between pain and sleep quality, function and posture in patients with FMS.^[23,24]^ With the decrease in pain, the vicious circle in which the patients are in may have been broken; accordingly, function, sleep quality and posture may have improved.

It was concluded that the conventional treatment consisting of TENS, therapeutic ultrasound, infrared and exercise therapy was effective on the evaluated parameters in this study. Vitorino et al found that conventional treatment and hydrotherapy methods applied to patients with FMS were effective on total sleep duration and quality of life.^[25]^ It is suggested that TENS produces endorphins by stimulating the sympathetic nervous system and brainstem nuclei and may prevent arthritis-related inflammation. Activity-related pain is a common feature in FMS^[18]^ and may be 1 reason why patients avoid activities and exercise that may increase pain. Pain is the most common indication for TENS. Success rates in clinical trials range from as low as 25% to as high as 80% to 95% and can be influenced by various factors.^[26]^ In addition, pulsed ultrasound therapy was found to improve sustained muscle contraction by increasing the permeability of the cell membrane; increases intracellular energy consumption; increases angiogenesis in the repair of ischemic tissues.^[26]^ A recent study by Almedia et al suggests that combined therapy with pulsed ultrasound and interferential current acting as an electrodiagnostic tool and physical therapy modality provides an effective pain treatment in FMS resulting in sleep improvement.^[27]^ The results of our study were in parallel with the literature.

This study concluded that the additional application of NMES caused additional changes in pain and posture. Although not statistically significant, the changes in sleep quality and function were greater. This may be due to the fact that NMES may be effective on pain inhibition through the mechanisms mentioned above. To the best of the authors’ knowledge, there are no studies investigating the effects of NMES in patients with FMS. This may be due to the presence of muscle damage in the etiology of FMS and discomfort during NMES application. However, with patient education, the problem of discomfort can be overcome and the benefits of NMES for pain inhibition can be seen. In addition, no complications developed in the patients who participated in this study. Therefore, it can be suggested that NMES is feasible and safe for FMS patients. In addition, improvements in posture may have occurred in the NMES group with additional pain inhibition. Bilek et al found that NMES applied in addition to conventional treatment was effective on posture in post stroke patients.^[28]^ Positive effects of NMES used in neurorehabilitation on posture and postural balance have been reported.^[29]^ This study was in parallel with the literature.

This study evaluating the effects of NMES on disease symptoms in patients with FMS had some limitations. The fact that we did not evaluate the muscle strength of the NMES-treated muscle can be seen as a limitation. In addition, the fact that we could not evaluate the posture assessment with a device such as posturography from which we could obtain more objective data can also be considered as a limitation. In addition, the lack of a group in which only NMES was applied to learn the effects of NMES alone may also be a limitation. Future studies may examine the isolated effects of NMES with its simple use.

## 7. Conclusion

This study was concluded that NMES applied in addition to conventional treatment provided significant changes in pain and posture compared to conventional treatment methods alone. Both treatment groups exhibited significant within-group improvements in Fibromyalgia Impact Questionnaire, visual analog scale, New York Posture Rating Chart, and PSQI scores posttreatment (*P* < .001 for most parameters), indicating the general efficacy of the interventions. However, the between-group comparisons highlight the added benefit of NMES in targeting pain and posture specifically. Although no complications were reported by participants during the intervention period, the safety profile of NMES should be interpreted with caution as side effects were not systematically evaluated. In addition, the absence of any complications or increase in pain in the individuals participating in the study may be interpreted that NMES is a safe and feasible method. It can be suggested that NMES can be applied in addition to routine treatment methods to achieve more success in the treatment of FMS. Further studies may examine the isolated effects of NMES in FMS patients.

## Author contributions

**Conceptualization:** Songul Baglan Yentur.

**Methodology:** Songul Baglan Yentur.

**Writing – original draft:** Muhammet Sahin Elbasti, Songul Baglan Yentur.

**Writing – review & editing:** Muhammet Sahin Elbasti, Songul Baglan Yentur.

## Supplementary Material


